# ILKAP, ILK and PINCH1 control cell survival of p53-wildtype glioblastoma cells after irradiation

**DOI:** 10.18632/oncotarget.5423

**Published:** 2015-10-07

**Authors:** Christina Hausmann, Achim Temme, Nils Cordes, Iris Eke

**Affiliations:** ^1^ OncoRay – National Center for Radiation Research in Oncology, Medical Faculty Carl Gustav Carus, Technische Universität Dresden, 01307 Dresden, Germany; ^2^ Section of Experimental Neurosurgery/Tumor Immunology, Department of Neurosurgery University Hospital Carl Gustav Carus, Technische Universität Dresden, 01307 Dresden, Germany; ^3^ Department of Radiation Oncology, University Hospital and Medical Faculty Carl Gustav Carus, Technische Universität Dresden, 01307 Dresden, Germany; ^4^ Helmholtz-Zentrum Dresden – Rossendorf, Institute of Radiooncology, 01328 Dresden, Germany; ^5^ German Cancer Consortium (DKTK), 01307 Dresden, Germany; ^6^ German Cancer Research Center (DKFZ), 69120 Heidelberg, Germany; ^7^ Radiation Oncology Branch, Center for Cancer Research, National Institutes of Health/National Cancer Institute, Bethesda, MD 20892, USA

**Keywords:** ILKAP, DNA repair, radioresistance, ILK, PINCH1

## Abstract

The prognosis is generally poor for patients suffering from glioblastoma multiforme (GBM) due to radiation and drug resistance. Prosurvival signaling originating from focal adhesion hubs essentially contributes to therapy resistance and tumor aggressiveness. As the underlying molecular mechanisms remain largely elusive, we addressed whether targeting of the focal adhesion proteins particularly interesting new cysteine-histidine-rich 1 (PINCH1), integrin-linked kinase (ILK) and ILK associated phosphatase (ILKAP) modulates GBM cell radioresistance. Intriguingly, PINCH1, ILK and ILKAP depletion sensitized p53-wildtype, but not p53-mutant, GBM cells to radiotherapy. Concomitantly, these cells showed inactivated Glycogen synthase kinase-3β (GSK3β) and reduced proliferation. For PINCH1 and ILKAP knockdown, elevated levels of radiation-induced γH2AX/53BP1-positive foci, as a marker for DNA double strand breaks, were observed. Mechanistically, we identified radiation-induced phosphorylation of DNA protein kinase (DNAPK), an important DNA repair protein, to be dependent on ILKAP. This interaction was fundamental to radiation survival of p53-wildtype GBM cells. Conclusively, our data suggest an essential role of PINCH1, ILK and ILKAP for the radioresistance of p53-wildtype GBM cells and provide evidence for DNAPK functioning as a central mediator of ILKAP signaling. Strategies for targeting focal adhesion proteins in combination with radiotherapy might be a promising approach for patients with GBM.

## INTRODUCTION

Glioblastoma multiforme (GBM) is the most common primary brain malignancy in adults [1]. Despite great efforts to optimize multimodal therapy regimens, the prognosis of patients with GBM is exceptionally poor due to the high invasive potential and the intrinsic therapy resistance of the tumor cells [1–5]. Therefore, clarifying molecular resistance mechanisms could prompt the development of new drugs and facilitate more effective treatment combinations.

In addition to a variety of well recognized gene mutations and epigenetic alterations in GBM [6], receptor tyrosine kinases, cytoplasmic protein kinases and integrins play an important role in resistance to radiotherapy in different tumor types [2, 7–14]. We recently reported a significant reduction in GBM cell radioresistance through targeting of β1 integrin [10], which facilitates cell binding to the extracellular matrix (ECM). However, the exact mechanism how integrin-mediated adhesion to ECM confers radioresistance to GBM and other cancer cells remains to be elusive.

A ternary protein complex composed of integrin-linked kinase (ILK), particularly interesting new cysteine-histidine-rich 1 (PINCH1) and parvin is key to integrin-mediated adhesion and the formation of focal adhesions [15]. While targeting of ILK results in radioresistance in head and neck and lung cancer cells and in radiosensitization of GBM cells [16–18], inhibition of PINCH1 causes radio- and chemosensitization in various tumor models [19, 20]. The unconventional structure of ILK that categorizes it as a pseudokinase [15] warranted further investigation to identify the role of ILK in signal transduction. One of its interacting partners is ILK-associated phosphatase (ILKAP). ILKAP has catalytic serine/threonine (S/T) phosphatase activity and belongs to the protein phosphatase 2C family [21]. In addition to its participation in cell cycling and apoptosis [21–23], ILKAP can shuttle between the cytoplasm and the nucleus using the importin nuclear trafficking system [24]. Further potential ILKAP-associated proteins include ribosomal S6 kinase 2 (RSK2), a signaling molecule of the MAPK pathway regulating invasion and cell motility [24, 25], and the tumor suppressor protein p53 [25].

p53 is frequently mutated in malignant tumors, where it is central to modifications in cell cycling, DNA repair and survival of cancer cells [26–30]. In response to cytotoxic stress, such as ionizing radiation, p53 is activated by ataxia telangiectasia mutated (ATM) to control cell cycling and to form a protein complex with DNA-dependent protein kinase (DNAPK) [26, 27]. Irradiation induces DNAPK autophosphorylation at T2609 and its participation in the repair of DNA double strand breaks (DSB) [31, 32]. Despite detailed insights into nuclear DNA repair processes, the mechanisms connecting integrin-associated signaling and cell adhesion-mediated radioresistance with DNA repair proteins like DNAPK and ATM remain unknown. Intriguingly, we recently reported that non-homologous end joining repair of DSB is conducted via the β1 integrin/FAK/JNK1 signaling axis in head and neck cancer cells [33].

In this study, we show that knockdown of PINCH1, ILK or ILKAP sensitizes p53-wildtype GBM cell lines to ionizing radiation. Mechanistically, ILKAP depletion results in p53 hyperphosphorylation and stabilization, DNAPK hypophosphorylation and increased DSB numbers after irradiation, indicating a crucial role of ILKAP for the cellular radiation response of human GBM cells.

## RESULTS

### PINCH1 and ILK were overexpressed in glioblastoma multiforme

**Figure 1:**
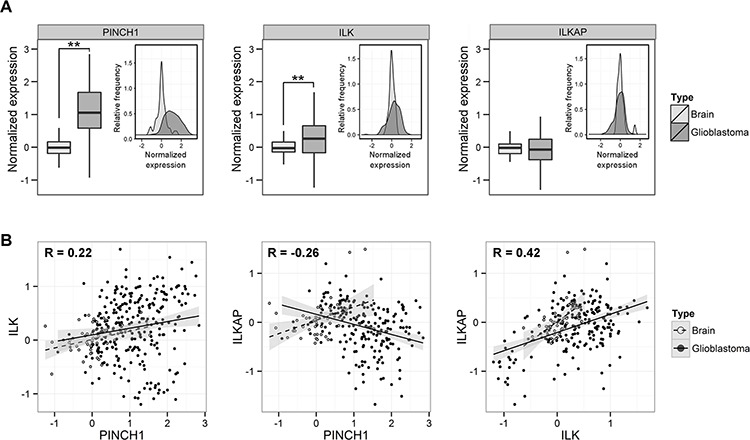
PINCH1 and ILK were overexpressed in glioblastoma multiforme **A.** Normalized protein expression (PINCH1, ILK, ILKAP) in normal brain and glioblastoma samples shown as histogram as a function of relative frequency and as box plot. Before analysis, values of each study were normalized to normal brain tissue samples. **B.** Data from ‘A’ depicted as dot blot graph. Correlation of expression of PINCH1 versus ILK, PINCH1 versus ILKAP and ILK versus ILKAP of glioblastoma tumors (solid line) and normal brain tissue (dashed line). (**P < 0.01).

Because PINCH1 and ILK are overexpressed in a variety of tumor entities [19, 34], we analyzed the mRNA expression in 757 samples of GBM and 127 samples of normal brain using the Oncomine data base (www.oncomine.org) (Supplementary Table S1). As shown in Figure 1, PINCH1 and ILK, but not ILKAP, were significantly overexpressed in GBM biopsies in comparison to normal brain tissue (Figure 1A). Interestingly, ILK and ILKAP expression levels strongly correlated (*R* = 0.42; *P* < 0.0001) in normal as well as malignant tissue (Figure 1B). Similar results were obtained for ILK and PINCH1 (*R* = 0.22; *P* < 0.0001), which were in line with other studies [35], while there was no significant association of PINCH1 and ILKAP expression (Figure 1B).

### Knockdown of integrin-associated proteins reduced survival and radioresistance of p53-wildtype glioblastoma cells

**Figure 2:**
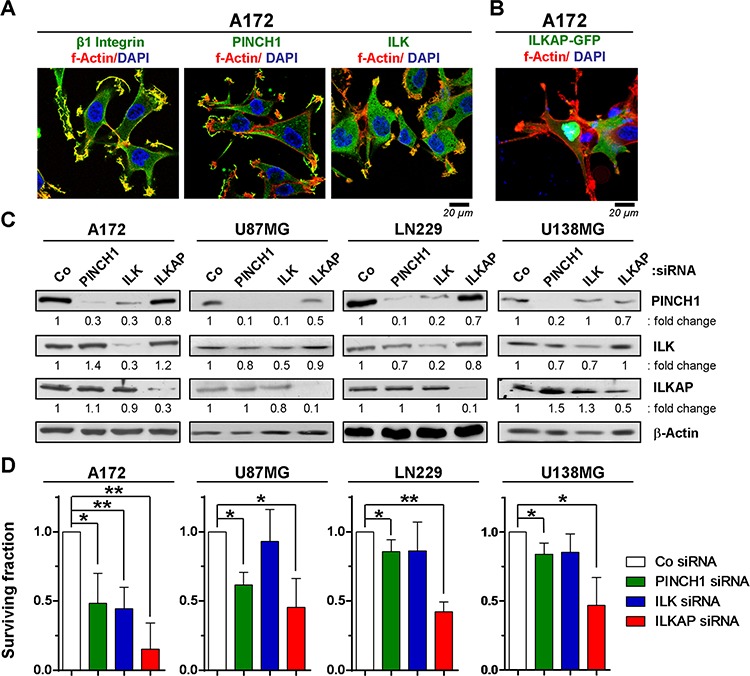
PINCH1 and ILKAP knockdown modulated clonogenic survival of glioblastoma cells **A.** Immunofluorescence staining of indicated proteins in A172 glioblastoma cells 24 h after plating. **B.** Immunofluorescence staining of A172 cells transiently transfected with an ILKAP-GFP construct. **C.** Western blot of whole protein lysates from siRNA-mediated A172, U87MG, LN229 and U138MG knockdown cell cultures and detection of PINCH1, ILK and ILKAP. β-Actin served as loading control. **D.** Clonogenic survival of PINCH1, ILK or ILKAP knockdown cell cultures. Data are mean ± SD (*n* = 3; *t*-test; *P < 0.05, **P < 0.01).

Targeting β1 integrin in glioblastoma cells enhances cellular radiosensitivity and hampers DNA repair [7, 10]. However, the molecular mechanisms and in particular the downstream molecules mediating these effects are widely unknown. We found that β1 integrin as well as the integrin-associated proteins PINCH1 and ILK were mainly located in focal adhesions of glioblastoma cells, whereas ILKAP was expressed in the cytoplasm and nucleus (Figure 2A and Figure 2B). Since the ILKAP antibody used for Western blot was not suitable for immunofluorescence, localization of ILKAP was revealed by transfection of an ILKAP-GFP construct. To analyze the impact of PINCH1, ILK and ILKAP on cell survival, we performed efficient siRNA-mediated depletion in four different glioblastoma cell lines (Figure 2C). While ILK silencing resulted in co-repression of PINCH1, PINCH1 knockdown had no effect on ILK expression (Figure 2C).

**Figure 3:**
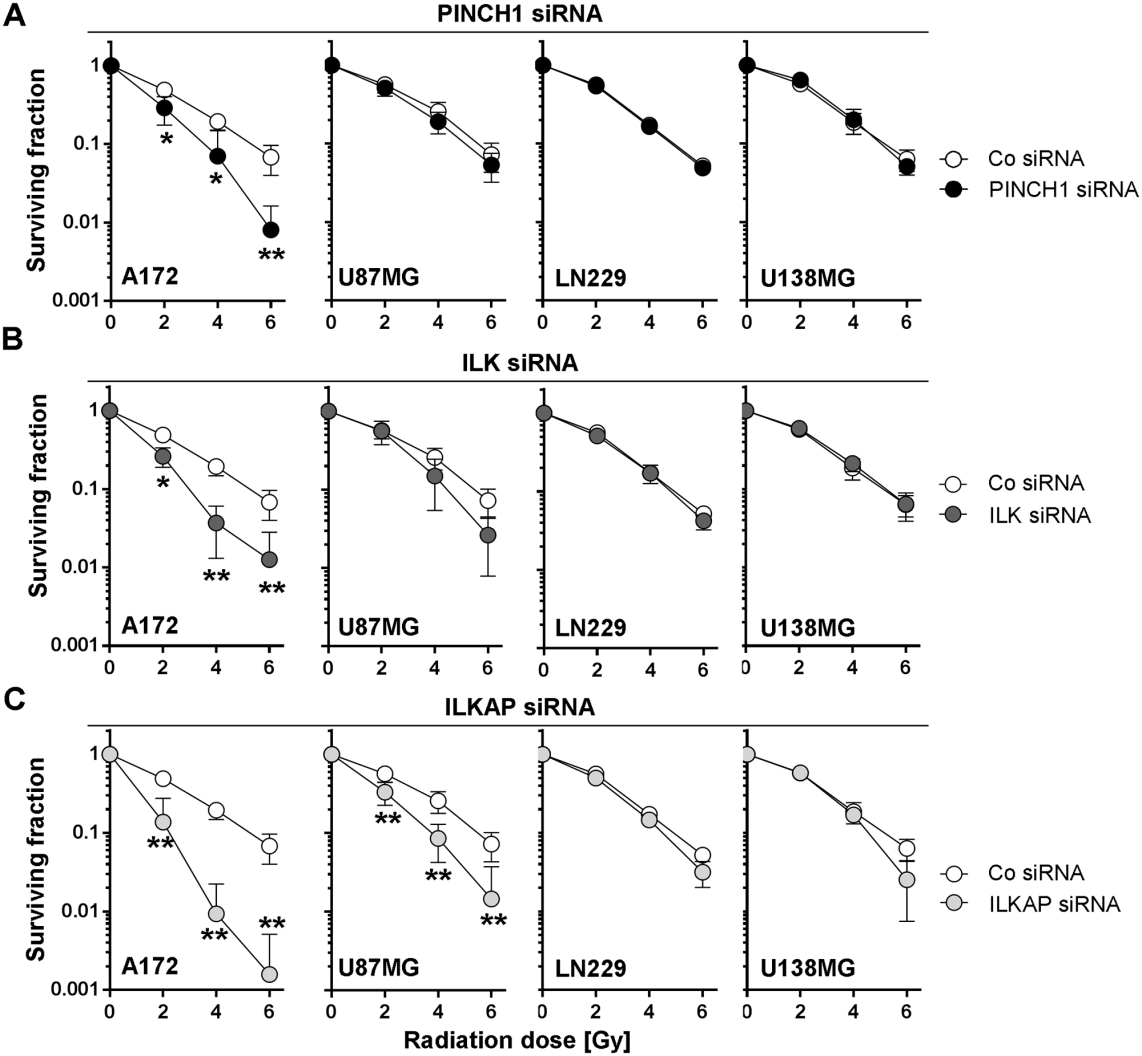
Depletion of PINCH1, ILK or ILKAP differentially radiosensitized human glioblastoma cell lines Clonogenic radiation survival of glioblastoma cells after **A.** PINCH1, **B.** ILK or **C.** ILKAP knockdown was measured by colony formation assays. Cells were irradiated 48 hours after siRNA transfection. Non-specific siRNA was used as control (Co siRNA). Data are mean ± SD (*n* = 3; *t*-test; *P < 0.05, **P < 0.01).

In p53-wildtype A172 and U87MG cells, knockdown of ILK, PINCH1 or ILKAP reduced basal survival (Figure 2D) and enhanced the radiosensitivity (Figure 3A–3C). In contrast, depletion of integrin-associated proteins failed to modify the cellular radiosensitivity of p53-mutant U138MG and LN229 cells (Figure 3A–3C).

### Depletion of PINCH1, ILK or ILKAP differentially affected DNA synthesis

**Figure 4:**
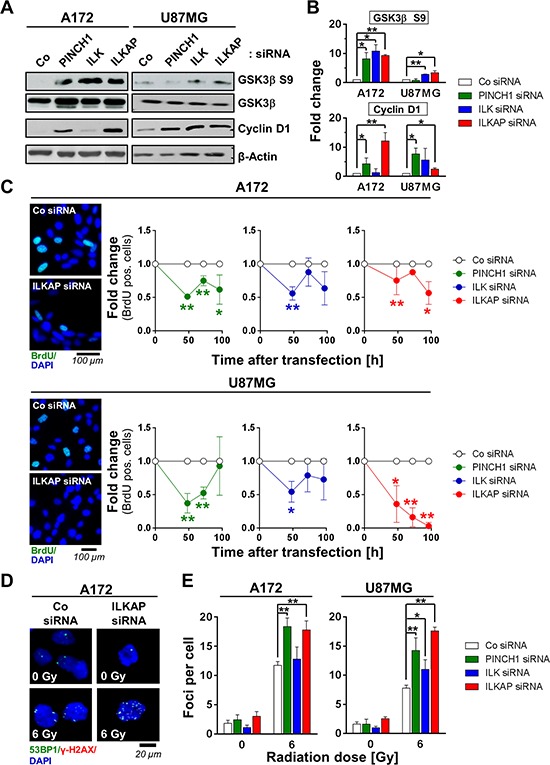
Integrin-associated proteins affected cell cycle and DNA double strand repair **A.** Western blot and **B.** densitometric analysis of p53-wildtype A172 and U87MG cells 48 h after PINCH1, ILK or ILKAP knockdown. Non-specific siRNA served as the control. **C.** Percentage of BrdU positivity as a marker for S-phase cells at different time points after siRNA transfection. Representative images are shown. **D.** Immunofluorescence staining of 53BP1 (green) and γH2AX (red) and **E.** quantitative analysis of foci numbers in irradiated A172 and U87MG cells after PINCH1, ILK or ILKAP depletion. Nuclei were stained with DAPI (blue). Data are mean ± SD (*n* = 3; *t*-test; *P < 0.05, **P < 0.01).

Because modulation of cell cycle distribution and DNA synthesis impact cellular radiosensitivity [36], we analyzed the effect of PINCH1, ILK and ILKAP inhibition on the cell cycle proteins Glycogen synthase kinase-3β (GSK3β) and Cyclin D1 as well as on the percentage of S-phase cells as a marker of proliferation. As shown in Figure 4, single downregulation of all three proteins significantly increased GSK3β S9 phosphorylation and Cyclin D1 expression in both A172 and U87MG glioblastoma cell lines (Figure 4A and Figure 4B). Exceptions were Cyclin D1 in ILK A172 knockdown cells and phospho-GSK3β S9 in PINCH1 U87MG knockdown cells.

Regarding BrdU incorporation as a proliferation index, we found the number of BrdU-positive cells decreased upon PINCH1, ILK and ILKAP depletion (Figure 4C). Importantly, while the effect of PINCH1 and ILK knockdown on the cell cycle was most pronounced after 48 h, ILKAP depletion elicited a continuous decline in the BrdU-positive cell compartment over the observation period of 100 h (Figure 4C).

### Knockdown of PINCH1, ILK and ILKAP impaired DSB repair

DSB have been shown to critically impact cell fate and radiation cell survival [37]. To evaluate if modulation of DNA repair is key to the observed radiosensitization, we examined the number of radiation-induced, γH2AX/53BP1-positive foci as a marker for DSB after inhibiting PINCH1, ILK or ILKAP. Strikingly, PINCH1 and ILKAP-depleted cells demonstrated significantly more γH2AX/53BP1-positive foci in 6-Gy irradiated cells compared with controls (Figure 4D and Figure 4E). ILK knockdown, however, only elicited elevated numbers of γH2AX/53BP1-positive foci in U87MG, but not in A172 cells (Figure 4E).

### Expression of wildtype p53 was essential for ILKAP-mediated radioresistance

**Figure 5:**
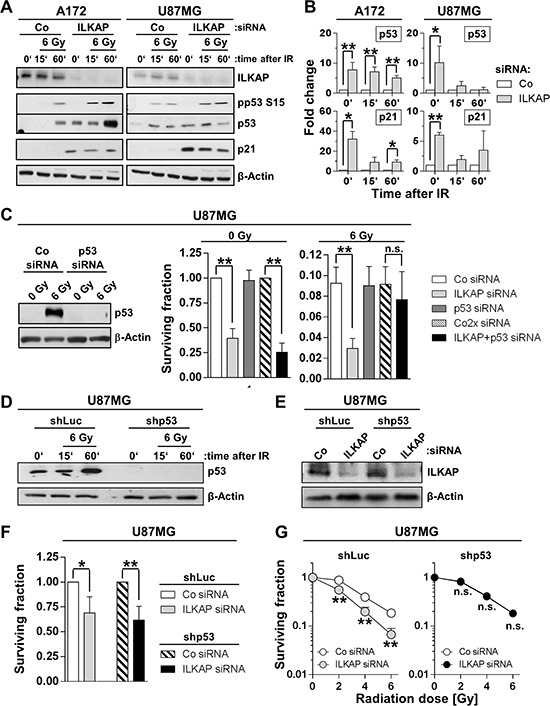
Expression of wildtype p53 was critical for the radiosensitizing effect upon ILKAP knockdown **A.** Protein expression and **B.** densitometric evaluation of ILKAP, p53, phospho-p53 (pp53) and p21 in p53 wildtype A172 and U87MG glioblastoma cells 15 and 60 minutes after irradiation with 6 Gy. Unirradiated cells were used as control. **C.** Basal and radiation survival of U87MG cells after p53 or ILKAP single knockdown and p53/ILKAP double knockdown. Efficacy of siRNA transfection was confirmed by Western blotting. Non-specific siRNA served as control (Co siRNA). Western blot for p53 **D.** and ILKAP knockdown **E.** and surviving fraction of **F.** unirradiated and **G.** irradiated U87MG stably transfected with shRNA directed against p53 (shp53). shRNA directed against luciferase (shLuc) was used as control. ILKAP knockdown was performed using siRNA (Co, non-specific siRNA control). Data are mean ± SD (*n* = 3; *t*-test; *P < 0.05, **P < 0.01).

To evaluate the role of p53 status in ILKAP-mediated radiosensitization, we analyzed the expression and phosphorylation of p53 and p21 after irradiation in ILKAP-depleted cells (Figure 5A and Figure 5B). In comparison with controls, ILKAP knockdown resulted in enhanced expression and stabilization of both p53 and p21 as well as p53 S15 hyperphosphorylation after radiation (Figure 5A and Figure 5B). Dual p53/ILKAP targeting abrogated the radiosensitization mediated by single ILKAP targeting (Figure 5C). These results were confirmed in U87MG cells with a stable shRNA-mediated p53 suppression (U87MG shp53) (Figure 5D–5G). Here, ILKAP knockdown only reduced radiation survival of U87MG luciferase shRNA expressing control cells (U87MG shLuc), but not p53-lacking U87MG shp53 cells (Figure 5G), indicating that p53 is an essential mediator of ILKAP-mediated radioresistance.

### ILKAP modulated the phosphorylation of DNAPK after irradiation

**Figure 6:**
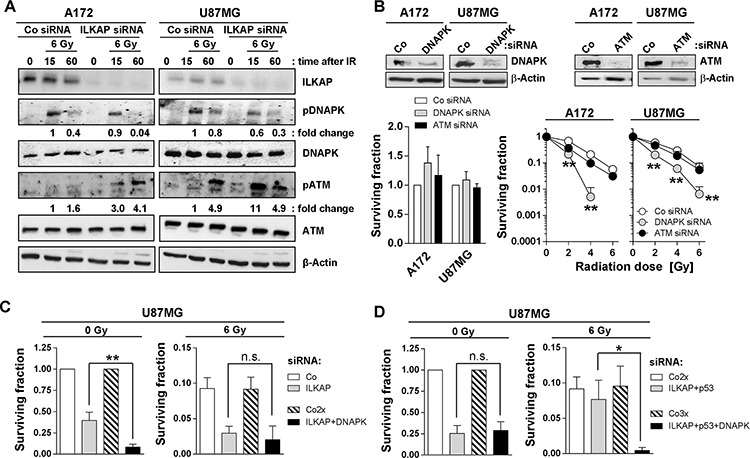
ILKAP modulated radiation-induced phosphorylation of DNA repair proteins **A.** Protein expression and phosphorylation of indicated proteins after ILKAP knockdown and irradiation (Co, non-specific siRNA control). β-Actin was used as loading control. Fold change was calculated by normalization of densitometric values to the level of the control siRNA cells 15 minutes after irradiation with 6 Gy. **B.** Basal and radiation survival of A172 and U87MG cells after siRNA mediated downregulation of DNAPK or ATM. Non-specific siRNA was used as control (Co siRNA). Knockdown efficiency is shown by Western blotting. Surviving fraction and radiosensitivity of U87MG cells after combined knockdown of **C.** ILKAP and DNAPK or **D.** ILKAP, p53 and DNAPK. Data are mean ± SD (*n* = 3; *t*-test; *P < 0.05, **P < 0.01).

Radiosensitization and enhanced levels of radiation-induced foci indicate a function of ILKAP in DNA repair. Interestingly, nuclear ILKAP expression increased after irradiation, indicating a cytoplasmic-nuclear shuttling of ILKAP (Supplementary Figure S1). After ILKAP knockdown, cells showed reduced DNAPK phosphorylation after irradiation, while ATM phosphorylation was increased (Figure 6A and Supplementary Figure S2). Both DNAPK and ATM have been reported to regulate radiation survival of tumor cells [27, 31, 38–40]. To assess a putative functional interaction between ILKAP and DNAPK or ATM, we performed single DNAPK or ATM knockdown and compared the radiosensitivity to cells with simultaneous knockdown of ILKAP. DNAPK and ATM silencing had no significant effect on basal glioblastoma cell survival (Figure 6B). However, radiosensitization occurred upon DNAPK but, surprisingly, not upon ATM depletion (Figure 6B). Combined ILKAP/DNAPK knockdown further reduced the surviving fraction of non-irradiated cells in comparison to ILKAP knockdown alone (Figure 6C). Radiation survival after ILKAP or ILKAP/DNAPK depletion was not significantly different, indicating that both proteins are part of the same signaling axis (Figure 6C).

To clarify the role of p53, we achieved p53/ILKAP/DNAPK triple knockdown and found this to have no impact on basal survival relative to p53/ILKAP depletion (Figure 6D). However, DNAPK knockdown on top of p53/ILKAP silencing restored the radiosensitizing potential of ILKAP targeting (Figure 6D). These data strongly suggest that all three molecules, ILKAP, p53 and DNAPK, interdependently and collectively control the radiosensitivity of p53-wildtype glioblastoma cells. Based on these data, we hypothesize that DNAPK functions downstream of ILKAP or p53 (Supplementary Figure S3).

## DISCUSSION

Identification of the molecular mechanisms underlying resistance is fundamental for GBM therapy optimization. In this study, we analyzed the role of PINCH1, ILK and ILKAP on the cellular radiation response of GBM cells. We found significant radiosensitization and DNA repair perturbation, particularly after siRNA-mediated knockdown of ILKAP. Mechanistically, we provide evidence for a mutual interplay between ILKAP, p53 and DNAPK, which is key for the radiation response of GBM cells.

Differential target expression in tumor versus normal tissues provides a therapeutic window for molecular-targeting strategies and might be used as a biomarker profile for personalized medicine. Both PINCH1 and ILK are upregulated in different tumor entities [19, 41, 42]. In line with previous studies, our analysis showed that PINCH1 and ILK were significantly overexpressed in glioblastoma biopsies compared with normal brain tissue. While the PINCH1 expression data in human cancers are largely homogeneous, ILK expression seems to be more dependent on the tissue of origin, the grade of cellular differentiation and the tumor type [43, 44]. PINCH1 and ILK are both part of a ternary protein complex, together with parvin, and are interdependently regulated [45]. Accordingly, our co-expression analysis revealed a correlation between PINCH1 and ILK mRNA levels as well as a downregulation of PINCH1 after ILK knockdown. Similar results were obtained for ILK and ILKAP. Surprisingly, PINCH1 depletion had no effect on ILK expression in our experimental setup. These results are different from the data of Fukuda and colleagues, which show diminished ILK protein levels after PINCH1 knockdown in HeLa cells [45]. We therefore conclude that the interdependence between PINCH1 and ILK is strongly influenced by tumor and tissue type.

Molecules involved in ECM adhesion have been shown to essentially control important cell functions, including tumor growth and cell survival [9, 11, 46–50]. Inhibition of integrins, the main ECM receptors, reduces tumor cell survival and sensitizes GBM cells to radiotherapy [7, 10]. Our model relies on two facts: (i) PINCH1 targeting is known to elicit radiosensitization in tumors other than GBM [19, 20] and (ii) ILK has been reported as antisurvival determinant in irradiated cells [17, 51]. In line with this knowledge, we found decreased basal and radiation survival of glioblastoma cells after PINCH1 depletion and far less after ILK targeting. In addition, we investigated the ILK interacting protein ILKAP as a target for GBM.

Interestingly, in light of the high p53 mutation frequency in GBM [52], radiosensitization was only achieved by PINCH1, ILK or ILKAP knockdown in the p53-wildtype cell lines A172 and U87MG. Because β1 integrin inhibition reduces radiation survival independent of p53 status [7, 10], the downstream signaling of integrins, in particular the function of ILK, PINCH1 and ILKAP for the integrin-mediated radioresistance, seems to be different between p53-wildtype and p53-mutated GBM cell lines. Focal adhesion kinase (FAK), which is a central mediator of integrin signaling in several tumor entities [48, 53], could play a significant role in this process. This hypothesis is supported by a recent study showing a radiosensitizing effect of pharmacological FAK inhibition in U138MG (p53-mutated), but not in A172 or U87MG (p53-wildtype) cells [54].

Knockdown of ILK, PINCH1 or ILKAP altered cell cycling, with ILKAP depletion exerting the most pronounced effect. This was in line with enhanced GSK3β S9 phosphorylation and Cyclin D1 expression. Similar results on GSK3β have been obtained in human embryonic kidney cells [21]. Concurrently, both PINCH1 and ILKAP depletion were accompanied by an increase in radiation-induced γH2AX/53BP1-positive foci, suggesting a potential role for these molecules in DNA repair processes.

Based on the most congruent data obtained for ILKAP targeting on GBM cell radiosensitization, we further examined the association between ILKAP and p53. First, the radiosensitization after ILKAP knockdown was diminished by p53 depletion, indicating that p53 functions as a downstream mediator of ILKAP. Second, ILKAP knockdown promoted enhanced p53 protein stabilization and radiation-induced phosphorylation at S15. ILKAP may act as a direct p53 dephosphorylator, as shown for other structurally similar PP2Cδ phosphatases [55]. This hypothesis is further supported by Højlys-Larsen and colleagues showing phospho-p53 S15 peptides in ILKAP affinity pulldowns, which points to a molecular interaction of these two proteins [25].

Since we not only observed reduced radiation survival, but also enhanced DSB after irradiation of ILKAP depleted cells, we analyzed phosphorylation kinetics of the important DNA repair proteins DNAPK and ATM. We found an ATM hyperphosphorylation at S1981 in the absence of ILKAP. These results are consistent with previous data indicating ATM as a potential target for ILKAP phosphatase activity [25]. As ATM has been shown to regulate DNAPK phosphorylation at T2609 [56], we were surprised to find an attenuated radiation-induced DNAPK phosphorylation after ILKAP silencing. One explanation for this could be that DNAPK phosphorylation at T2609 is regulated by more than one mechanism including a possible role of DNAPK itself by an autophosphorylation process [57, 58].

Although ATM is a known modulator of radiation survival [39, 40, 59], knockdown of ATM did not affect radiosensitivity in p53-wildtype glioma cells. Similar observations have been described by Biddlestone-Thorpe and colleagues. In this study, ATM inhibition preferentially sensitized p53-mutant glioblastoma cell lines but not p53-wildtype cells [60]. In contrast, DNAPK as well as combined ILKAP/DNAPK depletion led to significant radiosensitization which was comparable to the single ILKAP knockdown. Therefore we concluded that both proteins are part of the same signaling axis that regulates DNA repair and radiation resistance. Accordingly, the proper function of p53 after DNA damage is dependent on p53 interacting with DNAPK, suggesting a putative link between DNAPK and ILKAP/p53 signaling [26, 27]. As DNAPK knockdown also reduces the radiation survival of p53-depleted cells, DNAPK seems to be localized most downstream in this signaling pathway.

In summary, our study demonstrates an essential role for PINCH1, ILK and ILKAP in the radioresistance of p53-wildtype GBM cells. Mechanistically, the data presented provide evidence for DNAPK functioning as a central mediator of ILKAP signaling. Thus, strategies for targeting focal adhesion proteins in combination with radiotherapy might be a promising therapeutic approach for patients with GBM.

## MATERIALS AND METHODS

### Antibodies and reagents

Antibodies against DNAPK T2609 (cat.no. #ab18356)(Abcam, Cambridge, UK), PINCH1 (cat.no. #612711, Western blotting, Immunofluorescence), GSK3β (cat.no. #610202), p53 (cat.no. #610183), BrdU (cat.no. #347580), β1 integrin (cat.no. # 610468, Western blotting)(BD, Heidelberg, Germany), GSK3β Ser9 (cat.no. #9336), DNAPK (cat.no. #4602), ILK (cat.no. #3856, Western blotting, Immunofluorescence), ATM (cat.no. #2873), ATM S1981 (cat.no. #4526), p53 S15 (cat.no. #9284), Histone H3 (cat.no. #9715)(Cell Signaling, Frankfurt, Germany), ILKAP (cat.no. #07-712), γH2AX (cat.no. #05-636), β1 integrin (cat.no. #05-232, Immunofluorescence)(Upstate, Lake Placid, USA), 53BP1 (cat.no. #NB100-904)(Novus biologicals, Herford, Germany) and horseradish peroxidase-conjugated donkey anti-rabbit and sheep anti-mouse (Amersham, Freiburg, Germany), FITC anti-mouse (Dako, Hamburg, Germany), AlexaFluor 594 anti-mouse, AlexaFluor 488 anti-rabbit antibodies (Invitrogen, Darmstadt, Germany) were purchased as indicated. Complete protease inhibitor cocktail was from Roche (Mannheim, Germany). SuperSignal West Dura Extended Duration Substrate was from Thermo Scientific (Bonn, Germany). Nitrocellulose membranes were from Schleicher and Schuell (Dassel, Germany). Vectashield/DAPI mounting medium was purchased from Alexis (Gruenberg, Germany). Oligofectamine was purchased from Invitrogen and BrdU and BSA were purchased from Serva (Heidelberg, Germany).

### Cell culture and radiation exposure

Tumor cell lines A172, U138MG, U87MG and LN229 were obtained from the American Type Culture Collection (Manassas, VA, USA). Authenticated U87MG shp53 cells with stable RNAi of p53 and U87MG shLuc control have been described previously [61]. Cells were cultured in Dulbecco's Modified Eagle Medium (DMEM) containing Glutamax-I supplemented with 10% fetal calf serum and 1% non-essential amino acids (PAA, Cölbe, Germany) at 37°C in a humidified atmosphere containing 7% CO_2_. In all experiments, asynchronously growing cells were used. Irradiation was delivered at room temperature using single doses of 200 kV X-rays (Yxlon Y.TU 320; Yxlon, Copenhagen, Denmark) filtered with 0.5 mm Cu. The absorbed dose was measured using a Duplex dosimeter (PTW, Freiburg, Germany). The dose-rate was approximately 1.3 Gy/min at 20 mA and applied doses ranged from 0 to 6 Gy.

### mRNA analysis of human glioblastoma and normal brain tissue biopsies

PINCH1, ILK and ILKAP mRNA analysis was performed as recently published [19, 62]. Data were obtained from the Oncomine database (www.oncomine.org). The expression values of the different studies (Supplementary Table S1) were first normalized to the median of the expression levels in normal brain tissue. Distribution, expression and statistics were calculated from pooled data using R software version 3.1.0. For comparison, the Welch's *t*-test adaptation was used.

### siRNA transfection

PINCH1 siRNA (sequence: 5′-GGACCUAUAUG AAUGGUUUtt-3′), ILK siRNA (sequence: 5′-GGGCAAU GACAUUGUCGUGtt-3′), ILKAP siRNA (sequence: 5′-GGUUCUCUUGCCACAUCAAtt-3′), p53 siRNA (sequence: 5′-GGGUUAGUUUACAAUCAGCtt-3′), DNAPK siRNA (sequence: 5′- GGCAAUUCGUC CUCAGAUUtt-3′) and ATM siRNA (sequence: 5′-GG CACAAAAUGUGAAAUUCtt-3′) were obtained from Applied Biosystems (Darmstadt, Germany) and the non-specific control siRNA (sequence: 5′-GCAGC UAUAUGAAUGUUGUtt-3′) from MWG (Ebersberg, Germany). SiRNA delivery was performed as published [19]. Twenty-four hours after transfection with oligofectamine and 20 nM siRNA under serum-free conditions, cells were plated for colony formation assays, immunofluorescence staining or protein lysates.

### Colony formation assay

The colony formation assay was used to measure clonogenic cell survival and performed as recently published [10]. Cells were irradiated 24 h after plating. Cell colonies with a minimum of 50 cells were microscopically counted at 9 days (U87MG, LN229) or 12 days (A172, U138MG) after plating. Plating efficiencies were calculated as follows: numbers of colonies formed/numbers of cells plated. Surviving fractions (SF) were calculated as follows: numbers of colonies formed/(numbers of cells plated [irradiated] × plating efficiency [unirradiated]). Each point on the survival curves represents the mean surviving fraction from at least three independent experiments.

### Total protein extracts and western blotting

Adherent cells were rinsed with ice-cold 1XPBS prior to harvesting total proteins by scraping using modified RIPA buffer (50 mM Tris-HCl (pH 7.4), 1% Nonidet-P40, 0.25% sodium deoxycholate, 150 mM NaCl, 1 mM EDTA, Complete protease inhibitor cocktail (Roche), 1 mM Na_3_VO_4_, 2 mM NaF). Cells in suspension were centrifuged and washed with ice-cold 1XPBS. The supernatant was discarded and the remainder was lysed with modified RIPA buffer. Samples were stored at −80°C. Total protein amounts were measured via BCA assay (Pierce, Bonn, Germany). After SDS-PAGE and transfer of proteins onto nitrocellulose membranes (Schleicher and Schuell), probing and detection of specific proteins was accomplished with indicated antibodies and ECL as described [63].

### BrdU staining

To analyze the effect of PINCH1, ILK and ILKAP on cell cycle, the percentage of S-phase cells was determined after knockdown. Before fixation with 3% formaldehyde, cells were incubated with 10 μM BrdU for 10 min. After permeabilization with 0.25% Triton X-100/PBS for 10 min, preparation of samples with 1N HCl and 2N HCl for 10 min and blocking with 1% BSA/PBS, BrdU and Nuclei staining was accomplished with specific antibodies and Vectashield/DAPI mounting medium. Images were obtained with an Axioscope 2plus fluorescence microscope (Zeiss).

### Immunofluorescence staining

For localization of integrin-associated proteins, immunofluorescence staining was performed as previously described [19]. Cells were cultured for 24 h, fixed with 1% formaldehyde/PBS and permeabilized with 0.25% Triton X-100/PBS. Staining was performed with specific primary antibodies and fluorescence-labeled secondary antibodies. Nuclei were stained with Vectashield/DAPI mounting medium. Representative immunofluorescence images were obtained using a Laser Scanning Microscope LSM510 meta (Zeiss).

### ILKAP construct and plasmid transfection

The coding sequence of the human ILKAP gene was amplified by PCR from cDNA generated from human placental mRNA using specific primers (Fw: 5′-GGGGTACCATGGACC TCTTCGGGGACCTG -3′; Rev: 5′-CGGGATCC CGGTGGGGTATCCGCACC ACCAT-3′) and cloned into the pEGFP-N1 vector (Invitrogen) after restriction with *Kpn*I and *Bam*HI. Transient transfection was performed as published using Lipofectamine [19]. In brief, cells were plated 24 h prior to transfection. Plasmid and lipofectamine were incubated for 20 min at room temperature. Cells were washed 5 h after transfection. Immunofluorescence images were obtained with a Laser Scanning Microscope LSM510 meta.

### γH2AX/53BP1 assay

To provide further mechanistic insight into the enhanced radiosensitivity after PINCH1, ILK and ILKAP silencing, we measured residual DNA-double strand breaks (rDSB) by using the foci assay. As previously published [63], rDSBs were visualized by double staining of phosphorylated H2AX (γH2AX) plus p53 binding protein-1 (53BP1). Cells were fixed with 1% formaldehyde/PBS at 24 h after X-ray irradiation (0 or 6 Gy). Permeabilization with 0.25% Triton X-100/PBS preceded staining with specific anti-γH2AX and anti-53BP1 antibodies and Vectashield/DAPI mounting medium. γH2AX/53BP1-positive nuclear foci of at least 150 cells from three independent experiments were counted microscopically with an Axioscope 2plus fluorescence microscope (Zeiss) and defined as rDSBs.

### Protein fractionation

The Subcellular Protein Fractionation Kit for Cultured Cells (Pierce) was used according to the manufacturer's protocol and as published [64]. In brief, 24 h after plating cells were irradiated with 6 Gy or left unirradiated. At different time points after irradiation, cell lysis and fractionation were conducted. Equal loading was ensured by total protein concentration measurement using the BCA assay (Pierce). Fractionation efficacy was confirmed with detection of β1 integrin for the membrane fraction and Histone H3 for the nuclear fraction.

### Data analysis

Means ± SD of at least three independent experiments were calculated with reference to untreated controls defined in a 1.0 scale. To test statistical significance, a Student's *t*-test was performed using Microsoft^®^ Excel 2003. Results were considered statistically significant if a *P*-value of less than 0.05 was reached. Densitometry of Western blots was performed by scanning of the exposed film and using ImageJ analysis software (http://www.nih.gov).

## SUPPLEMENTARY FIGURES AND TABLE


