# Supplementary material for: ILKAP, ILK and PINCH1 control cell survival of p53-wildtype glioblastoma cells after irradiation
Source: Oncotarget. 2015 Oct 7;6(33):34592–605. doi: 10.18632/oncotarget.5423 (PMC4741475; doi:10.18632/oncotarget.5423)
Supplement: Supplementary file 1 [file oncotarget-06-34592-s001.pdf]

SUPPLEMENTARY FIGURES AND TABLE

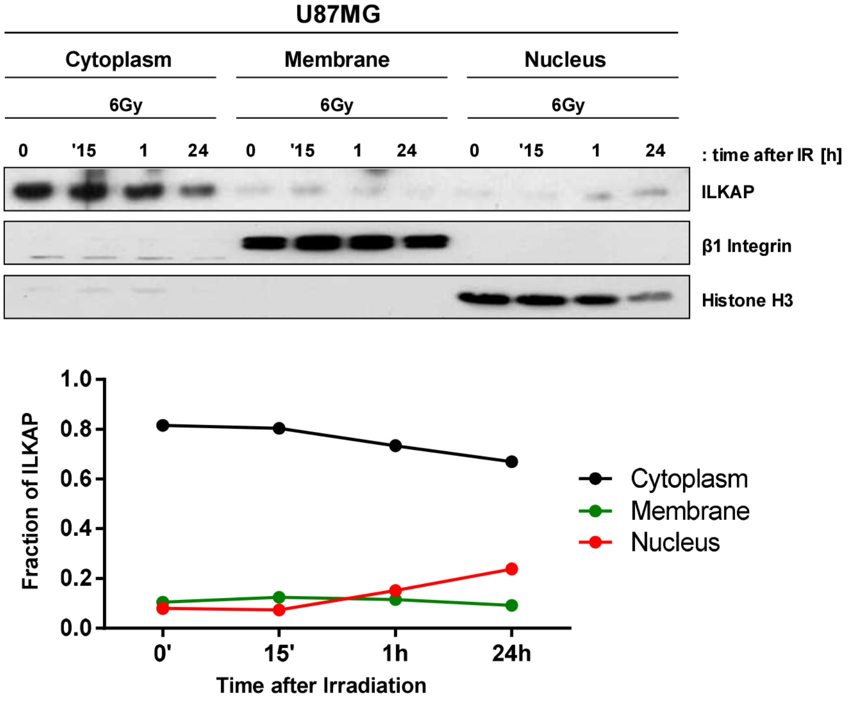

Supplementary Figure S1: ILKAP translocated to the nucleus after irradiation. For protein fractionation experiments, cells were plated 24 h prior to irradiation. At the indicated time points after irradiation, cells were lysed as described. Non-irradiated cells were used as controls. Detection of D1 integrin and Histone H3 were performed to confirm efficacy of fractionation. Western blots were evaluated using densitometric analysis.

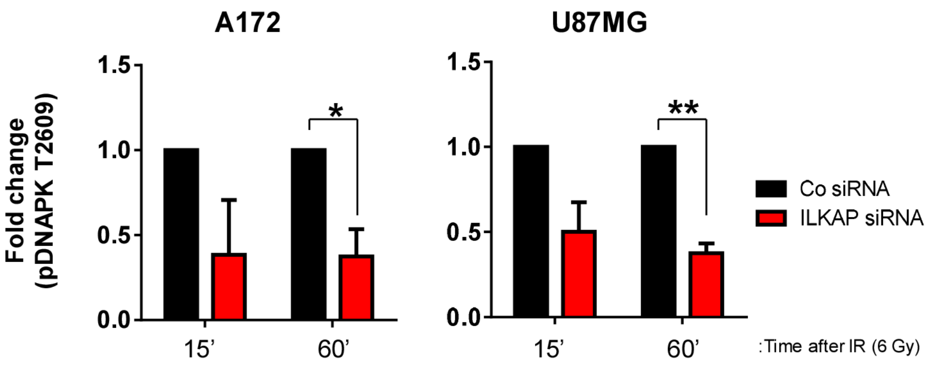

Supplementary Figure S2: ILKAP deletion reduced radiation-induced DNAPK phosphorylation. Densitometric analysis of Western blots in A172 and U87MG glioblastoma cells at indicated time points after irradiation with 6 Gy. ILKAP knockdown was performed with specific siRNA. Non-specific siRNA was used as control. Data are mean ± SD (*n* = 2; t-test; $*P < 0.05$, $**P < 0.01$).

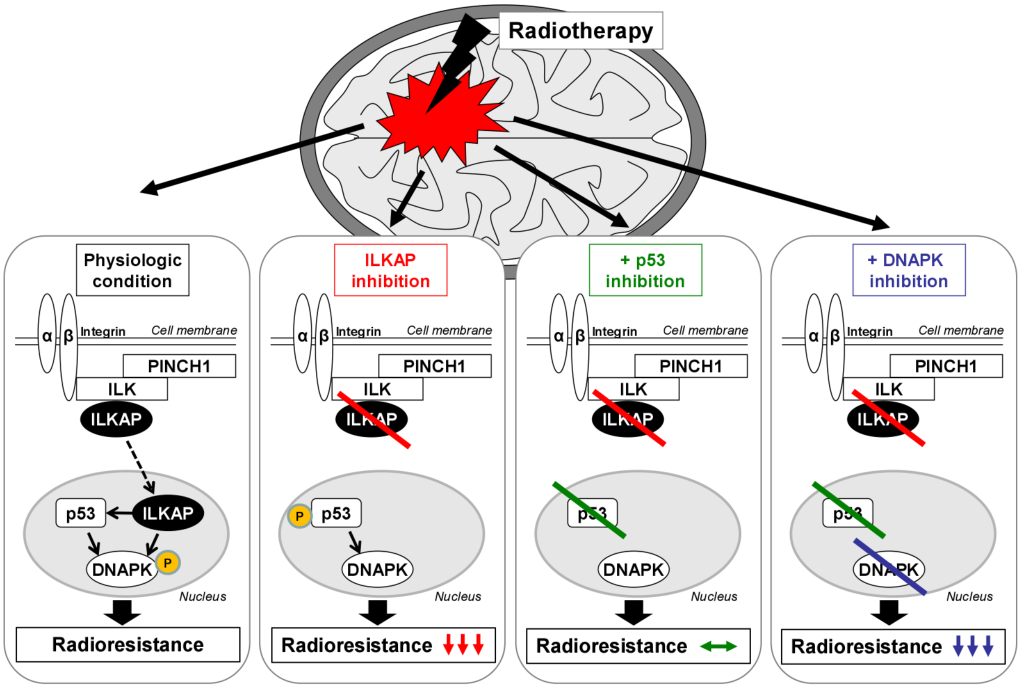

Supplementary Figure S3: Schematic of ILKAP, p53 and DNAPK regulation of radioresistance in p53-wildtype glioblastoma cells. Under physiologic conditions integrin-associated signaling confers radioresistance by regulating the DNA repair protein DNAPK. ILKAP knockdown results in radiosensitization, whereas a combined inhibition of ILKAP and p53 has no effect on cellular radiosensitivity. Additional targeting of DNAPK restores the radiosensitizing effect, which can be observed after single ILKAP inhibition.

Supplementary Table S1: List of Data sets included in Microarray analysis of *PINCH1, ILK* and *ILKAP* mRNA expression using the oncomine database (www.oncomine.org)

	Data sets/Publications
**1.**	Sun L, et al. (2006) Neuronal and glioma-derived stem cell factor induces angiogenesis within the brain. Cancer Cell. 9(4):287–300.
**2.**	Bredel M, et al. (2005) Functional network analysis reveals extended gliomagenesis pathway maps and three novel MYC-interacting genes in human gliomas. Cancer Res. 65(19):8679–89.
**3.**	Shai R, et al. (2003) Gene expression profiling identifies molecular subtypes of gliomas. Oncogene. 22(31):4918–23.
**4.**	Liang Y, et al. (2005) Gene expression profiling reveals molecularly and clinically distinct subtypes of glioblastoma multiforme. Proc Natl Acad Sci U S A. 102(16):5814–9.
**5.**	Murat A, et al. (2008) Stem cell-related “self-renewal” signature and high epidermal growth factor receptor expression associated with resistance to concomitant chemoradiotherapy in glioblastoma. J Clin Oncol. 26(18):3015–24.
**6.**	Lee J, et al. (2006) Tumor stem cells derived from glioblastomas cultured in bFGF and EGF more closely mirror the phenotype and genotype of primary tumors than do serum-cultured cell lines. Cancer Cell. 9(5):391–403.
**7.**	National Cancer Institute, National Institutes of Health, Bethesda, MD 20892 (2013) The Cancer Genome Atlas - Glioblastoma Multiforme Gene Expression Data. *http://tcga-data.nci.nih.gov/tcga/*
